# Prevalence and Antimicrobial Susceptibility Patterns of ESBL, AmpC and Carbapenemase-producing *Enterobactericeae* Isolated from Hospitalized Patients in Azerbaijan, Iran

**Published:** 2018

**Authors:** Reza Ghotaslou, Mohammad Reza Sadeghi, Mohammad Taghi Akhi, Alka Hasani, Mohammad Asgharzadeh

**Affiliations:** a *Immunology Research Center, Tabriz University of Medical Sciences, Tabriz, Iran. *; b *Department of Microbiology, School of Medicine, Tabriz University of Medical Sciences, Tabriz, Iran.*; c *Department of Laboratory sciences, Islamic Azad University, Urmia branch, Urmia, Iran. *; d *Department of Laboratory Sciences, Paramedical Faculty and Biotechnology Research Center, Tabriz University of Medical Sciences, Tabriz, Iran.*

**Keywords:** Antibiotic resistance, β-lactamase-producing *Enterobactericeae*, Hospitalized patients, Azerbaijan, Iran

## Abstract

The current study was conducted to determine prevalence and antimicrobial susceptibility patterns, ESBL, AmpC and carbapenemase- producers among clinical isolates of *Enterobacteriaceae*. Three hundred and seven non-duplicative clinical isolates were collected from hospitalized patients in five medical centers in Azerbaijan, Iran. Initial screening for β-lactamase production was performed using disc agar diffusion according to Clinical Laboratory Standards Institute (CLSI) guidelines. Phenotypic confirmatory tests was done using total ESBL/AmpC confirm kit, KPC/MBL and OXA-48 confirm kit according to manufacturer’s instructions. The overall prevalence of ESBL, AmpC, and carbapenemase-producing strains were 42.7% (131/307), 14.0%(43/307) and 4.9% (15/307), respectively. The prevalence of ESBLs was 38.35%, 64.9% and 35.7% for *E. coli*, *K. pneumoniae* and *E. cloacae,* respectively. Carbapenemase activity was only observed among 15 *K. pneumoniae* isolates and detected phenotypes include MBL (9/57, 15.8%), KPC (4/57, 7.0%), and OXA-48 (2/57, 3.5%). Fourteen out of 307 isolates (4.6%) were recognized to have ESBL or AmpC hyper-producer with decreased cell wall permeability phenotype. All 4 shigella strains were positive for ESBL and 4 isolates of *P. mirabilis*, 2 isolates of *M. morganii* and 1 seratia spp. were detected as AmpC producer. The only *C. freundii* strain isolated was positive for both ESBL and AmpC. This study reveals high prevalence of multidrug-resistant (MDR) β-lactamase-producing *Enterobactericeae* reaching 89.5%. Imipenem and meropenem showed potent antibacterial activities against all MDR β-lactamase-producers except for carbapenemase producers. After carbapenems, amikacin, piperacillin/tazobactam and amoxicillin/clavulanic acid were second the most effective drugs against β-lactamase-producing *E. coli* isolates.

## Introduction

Beta-lactamase production has been known as the main mechanism for β-lactam drugs resistance among members of the family *Enterobactericeae*. Resistance to different β-lactam agents such as penicillins, cephalosporins, monobactams and carbapenems have been mostly attributed to the presence of several types of enzymes, including ESBLs (1), Ambler class C cephalosporinases (2), and carbapenemases (3). ESBLs confer resistance to broad-spectrum cephalosporins, which are the antibiotics that primarily administrated for treatment of infections caused by *Enterobactericeae*. In past two decades, widespread dissemination of ESBL-producing *E. coli*, *K. pneumoniae* and Enterobacter spp. has been associated with complicated community and hospital-onset infection with poor clinical outcomes (4-5). Additionally, these strains are often resistant to other classes of antimicrobial agents, such as fluoroquinolones, trimethoprime-sulfamethoxazole and aminoglycosides (6-7). Ambler class C cephalosporinases (AmpCs) render *Enterobactericeae* resistant to most β-lactams except for cefepime and carbapenems. Although most of the AmpCs are inducible and chromosomally encoded enzymes, some of them are constitutively expressed plasmid-mediated AmpCs that have originated from mobilization of chromosomal AmpC genes by mobile integrative and conjugative elements (2). Acquired plasmid-mediated AmpC β-lactamases have been increasingly reported among members of family *Enterobactriaceae*, mainly due to the dynamic nature of these elements (2, 8-9). Because of poor response to broad-spectrum cephalosporins associated with ESBL and AmpC-producing organisms, carbapenems have been extensively used as drug of choice in treating infections particularly those caused by multi-drug resistant (MDR) Gram-negative bacteria (5, 10). However, emergence of novel β-lactamases have compromised effectiveness of carbapenem antibiotics in clinical settings (10-11). Resistance to carbapenems is usually mediated by mobile elements-encoded carbapenemases belonging to class A (KPC and GES), class B (MBLs) and class D (OXA-48) of ambler classification (12-14) and has been increasingly described among *Enterobactericeae* family (3, 15). Moreover, resistance to carbapenems related to ESBL or AmpC hyper production along with decreased permeability (porin loss) is prevalent in this family. Available treatment options have been strongly limited due to pan-resistance of carbapenemase-producing strains to almost all β-lactam antibiotics (16-17). ESBL, AmpC as well as carbapenemase-producing strains give rise to morbidity and mortality (18). Little information was available about the accurate prevalence of ESBL, AmpC and carbapenemase-producing *Enterobacteriaceae* in Iran. The aim of this study was to determine prevalence and antimicrobial susceptibility patterns of ESBL, AmpC and carbapenemase-producing *Enterobacteriaceae* (CPE) isolating from hospitalized patients in five medical centers during 2014 in Azerbaijan, Iran. 

## Experimental

*Materials and Methods*

*Bacterial isolates*

A total of 307 non-duplicate clinical isolates of *Enterobacteriaceae* recovered from various clinical specimens (urine, blood, CSF, wound, burn and surgical wounds, tracheal and bronchial secretions, abdominal fluid, stool and sputum) from five medical centers of East and West Azerbaijan were included during January-July 2014. The clinical significance of all isolates was determined at each inpatient ward based on the patient’s compatible clinical and laboratory findings. Patient’s specific data including age, gender, and ward were recorded for all patients. Bacterial identification was performed to the species level using standard biochemical methods and confirmed by the API20E enteric identification system (bioMe´rieux, Marcy l’ Etoile, France).

*Phenotypic screening and confirmatory tests*

Initial screening for ESBL, AmpC, and carbapenemase production was performed based on the disc diffusion method using ceftazidime (30 µg), cefotaxime (30 µg), ceftriaxone (30 µg), cefoxitin (30 µg), imipenem (10 µg), meropenem (10 µg) and ertapenem (10 µg) discs (Rosco diagnostica Neo-sesitabs discs, Denmark) according to the Clinical Laboratory Standards Institute (CLSI) screening criteria for β-lactamase production (19). Non-susceptible strains to cefoxitin were considered presumptive AmpC producers. Suspected strains for β-lactamase production were further confirmed using total ESBL/AmpC confirm kit and KPC/MBL and OXA-48 confirm kit (Rosco diagnostica, Denmark). Since the detection of ESBLs can be obscured by chromosomal, AmpC producers, ESBL confirm kit (Rosco diagnostica, Denmark) was used to detect ESBLs in such isolates. The results were interpreted according to the CLSI guidelines (19) and manufacturer’s recommendations as follows: the presence of ESBL/AmpC activity was indicated by a ≥ 5 mm increase in zone diameter for either ceftazidime or cefotaxime in combination with both clavulanate and cloxacillin compared to either drug in combination with only cloxacillin or clavulanate for confirmation of ESBL or AmpC, respectively. ESBL activity in isolation with chromosomal AmpC was demonstrated by an increase of inhibitory zone of at least 5 mm when cefepime in combination with clavulanate was compared with only cefepime. Production of KPC and MBL was detected if inhibition zones around meropenem discs containing phenylboronic acid (KPC inhibitor) or dipiculinic acid (MBL inhibitor) were extended by more than 4 and 5 mm, respectively when compared to meropenem disc without inhibitor. Carbapenem resistance associated with AmpC production couple to decreased permeability was characterized by a ≥ 5 mm difference in zones between meropenem and meropenem/cloxacillin discs along with at least a 4 mm difference between meropenem and meropenem/phenylboronic acid discs. Temocillin resistant and susceptible isolates showing negative results of all synergy tests were identified as OXA-48 and porin-deficient ESBL producers respectively.

*Antimicrobial susceptibility testing*

Antimicrobial susceptibility of all β-lactamase-producing strains was determined by the disc diffusion method according to the CLSI criteria (19). Susceptibility of isolates was determined against cefepime (30 µg), ampicillin (10 µg), amoxicillin/clavulanic acid (20/10 µg), piperacillin/tazobactam (100/10 µg), ciprofloxacin (5 µg), gentamicin (10 µg), amikacin (30 µg) and trimethoprim-sulfamethoxazol (1.25/23.75 µg) discs (Rosco diagnostica, Denmark). *E. coli* ATCC 25922 and *K. pneumoniae* ATCC 700603 were used as quality control strains in each set of susceptibility tests.

*Statistical analysis*

Statistical analysis for descriptive statistics, including frequencies and cross tabulation of clinical, microbiological and epidemiological characteristics was done using the computer software program SPSS, version 21. The chi-squared test and Fisher’s exact test were used to determine the association between the β-lactamase production and MDR pattern. A p-value of ≤ 0.05 was considered as statistically significant.

## Results and Discussion

During January to July 2014, a total of 307 clinical isolates of *Enterobacteriaceae* were isolated from admitted patients in various hospital wards, including internal wards (n = 181, 59%), surgery (n = 55, 17.9%), intensive care units (n = 37, 12.1%), pediatric (n = 19, 6.2%) and burn (n = 15, 4.9%). The mean age of patient was 52 ± 22 years and 59.3% were females. The bacterial strains isolated included, *E. coli *(n = 219, 71.3%), *K. pneumoniae* (n = 57, 18.6%), *E. cloacae* (n = 14, 4.6%), *P. mirabilis* (n = 5, 1.6%), *K. oxytoca*, *M. morganii*, *S. flexneri*, *S. sonnei*, *P. vulgaris* (two isolates each), *S. marcescens *and *C. freundii* (one isolate each). The majority of isolates were predominantly obtained from urine samples (n = 219, 71.3%) while other recovered from blood cultures (n = 43, 14%), burn wound exudates (n = 13, 4.2%), wound purulent discharges (n = 11, 3.6%), tracheal and bronchial secretions (n = 7, 2.3%), sputum (n = 5, 1.6%), stools (n = 4 pathotype strains, 1.3%), abdominal fluid (n = 3, 1%) and cerebro-spinal fluid (n = 2, 0.7%). *E. coli* was the most common isolate from wards except for burns where *K. pneumoniae* was the most prevalent isolate. 

Based on the initial screening results for possible ESBL, AmpC and carbapenemase production, of total isolates (52.8%) met the criteria and were selected for further confirmatory tests. Overall prevalence of ESBL, AmpC, and carbapenemase-producing strains were 42.7% (131/307), 14% (43/307) and 4.9% (15/307), respectively. Of these, 67.2% (88/131) and 13% (17/131) of ESBL-producing strains, 74.4% (32/43) and 11.6% (5/43) of AmpC-producing strains and 40% (6/15) and 13.3% (2/15) of carbapenemase-producing strains were isolated from urine and blood samples, respectively. The source of remaining carbapenamase-positive strains was burn [four strains (26.7%)], wound [two strains (13.2%)] and sputum [one strain (6.7%)]. The highest prevalence of ESBLs was observed for internal ward (78/131, 59.5%), followed by surgery (21/131, 16%) and ICU (15/131, 11.4%). The AmpC-producing strains were predominantly obtained from internal wards (25/43, 58.1%) followed by surgery (11/43, 27.9%) and ICU (4/43, 9.3%). Among 307 isolates, 38.35% (84/219), 64.9% (37/57) and 35.7% (5/14) of *E.coli*, *K. pneumoniae* and *E. cloacae *isolates were found to be ESBL producers, respectively. These results for AmpCs include 10.5% (23/219) of *E. coli* and all isolates (100%) of *E. cloacae. *Nine strains out of 219(4.2%) *E.coli* isolates were positive for both ESBL and AmpC. AmpC activity was not detected among *K. pneumoniae* strains based on the phenotypic confirmatory tests. Carbapenemase activity was only observed among fifteen *K. pneumoniae* strains, detected phenotypes including MBL (9/57, 15.8%), KPC (4/57, 7%), and OXA-48 (2/57, 3.5%). In the current study, all four KPC-producing *K. pneumoniae* strains were isolated from patients hospitalized in burns (n = 2), surgery (n = 1) and internal (n = 1) wards of Sina hospital of Tabriz. Five out of nine MBL positive strains were isolated from internal (n = 4) and surgery (n = 1) wards of Imam Reza hospital of Tabriz and remaining four strains were isolated from Sina hospital of Tabriz (one isolate from of each ICU, surgery and burns) and Imam Khomeini hospital of Urmia (one Isolate from ICU). We also detected two ESBL positive, OXA-48 producing *K. pneumoniae* strains from internal and burn wards of Sina hospital.

Of 307 isolates 14(4.6%) were recognized to have ESBL/AmpC hyper producer with decreased cell wall permeability phenotypes based on non-susceptibility to carbapenem (particularly for ertapenem as intermediate), positive ESBL/AmpC confirmatory tests, and carbapenemase confirmatory results. Results of the initial screening and *in-vitro *antimicrobial susceptibility patterns of β-lactamase-producing strains are shown in Table 1 and 2, respectively.

Initial screening for β-lactamase production showed that 45.9% (141/307) of all isolates were ESBL positive. Based on the phenotypic confirmatory tests 42.7% of isolates were ESBL producer. Meropenem, imipenem, amikacin and, ertapenem were the most effective drugs against ESBL/AmpC producers with 91.8%, 91.2%, 88.6%, and 86.8% susceptibilities respectively. The rates of susceptibility against meropenem, imipenem, and ertapenem among all isolates were 95.1%, 94.7%, and 92.5%, respectively. Also piperacillin/tazobactam had good activity with susceptibility rate reaching 71%. On the other hand, susceptibility testing results showed moderate resistance to gentamicin, cefepime, and amoxicillin/clavulanic acid among ESBL/AmpC producers. High rates of resistance were observed against ampicillin, co-trimoxazole, and ciprofloxacin. The prevalence of multidrug resistant β-lactamase-producing isolates was 89.5% based on resistance against more than two antimicrobial classes.

Our knowledge about the occurrence and mechanisms of resistance to antimicrobial agents effectively help to combat drug resistant infections. The current study describes the rates and antimicrobial susceptibility patterns of ESBLs, AmpCs, and carbapenemase-producing members of family *Enterobacteriaceae* isolated from various clinical specimens in five major medical centers in East and West Azerbaijan, Iran. In the present study, overall prevalence of ESBLs was 42.7% and the rate of ESBLs among *K. pneumoniae *(64*.*9%) was more than *E. coli* (38.3%). The comparison of our findings with the respective data reported in the global surveillance studies including Tigecycline Evaluation and Surveillance Trial (TEST) and Meropenem Yearly Susceptibility Test Information Collection (MYSTIC) indicated that the prevalence of ESBL producers among *E. coli* and *K. pneumoniae* isolates was significantly higher in Iran compared to other parts of the world (20). Based on the data reported in TEST surveillance program in 2006 the rates of ESBL-producing *E. coli* and *K. pneumoniae* isolates were highest in Latin America (13.5% and 44%) followed by Asia/Pacific Rim (12% and 22.4%), Europe (7.6% and 13.3%) and north America (2.2% and 7.5%), respectively (20). According to MYSTIC program, the overall occurrence of ESBL-producing *Enterobacteriaceae* in Europe in 2006 was 5.6%. By species, it was 8.2% in *E. coli* and 9.8% in *Klebsiella* spp. (21). These values are considerable lower than those reported in the current study.

 In our study, the prevalence of resistance to third-generation cephalosporins among invasive isolates (isolates from blood and CSF) of *E. coli* and *K. pneumoniae* was 34.9% and 87.5%, respectively. Comparing these values with the data from recent European Antimicrobial Resistance Surveillance Network (EARS-Net) in 2011 (ranging from 3% to 22% for *E. coli* and 2.3% to 60.6% for *K. pneumoniae*) explains the high rates of ESBLs among this isolates in Iran. In this respect, our results are only in accordance with data from Slovakia (31%) and Cyprus (36.2%) for *E. coli* and those reported from Greece (75.8%) and Bulgaria (81%) for *K. pneumoniae *(22). The main reasons for high occurrence of ESBLs in Iran may be associated with self-medication and overuse of third-generation cephalosporins in hospital settings. Moreover, reported data in this study are relatively lower than those reported by recent studies in neighboring countries such as Turkey (50% and 80%), Pakistan (72% and 66%) and India (57% and 67%) for *E. coli* and *K. pneumoniae*, respectively (23-25).

**Table 1:** The antimicrobial susceptibility patterns of *Enterobacteriaceae* isolates from various clinical settings.

		Resistance to antimicrobial agents (%)																				
Wards (no)	Isolates (no; %)	CTX			CTR			CAZ			CFO			IMP			ETP			MRP		
		S	I	R	S	I	R	S	I	R	S	I	R	S	I	R	S	I	R	S	I	R
ICU (37)	*E. coli *(24; 64.9)	62.5	4.2	33.3	62.5	4.2	33.3	79.2	8.3	12.5	100			100			100			100		
	*K. pneumoniae *(10; 27.0)	40.0		60.0	40.0		60.0	40.0		60.0	80.0		20.0	80.0		20.0	70.0	10.0	20.0	80.0		20.0
	*E. cloacae *(3; 8.1)	66.7		33.3	66.7		33.3	100			33.3		66.7	100			100			100		
																					
Internal (181)	*E. coli *(144; 79.6)	54.9	0.7	44.4	53.5	0.7	45.8	62.5	8.3	29.2	82.6	2.8	14.6	100			97.9	1.4	0.7	99.3	0.7	
	*K. pneumoniae *(25; 13.8)	24.0		76.0	24.0		76.0	24.0	12.0	64.0	72.0	8.0	20.0	72.0	8.0	20.0	72.0	4.0	24.0	76.0		24.0
	*E. cloacae *(3; 1.7)	33.3		66.7	33.3		66.7	33.3		66.7			100	100			66.7		33.3	100		
	*P. mirabilis *(3; 1.7)	100			100			100			100			66.7	33.3		100			100		
	*P. vulgaris *(1; 0.6)	100			100			100			100			100			100			100		
	*K. oxytoca *(1; 0.6)	100			100			100			100			100			100			100		
	*S. flexneri *(1; 0.6)			100			100	100			100			100			100			100		
	*S. marcescens *(1; 0.6)	100			100			100			100			100			100			100		
	*k. pneumonia *(25; 13.8)																					
	*M. morganii *(1; 0.6)	100			100			100			100			100			100			100		
	*C. freundii *(1; 0.6)	100			100			100					100	100			100			100		
																						
Surgery (55)	*E. coli *(37; 67.3)	51.4	2.7	45.9	48.6	2.7	48.6	59.5	2.7	37.8	91.9	2.7	5.4	100			100			100		
	*K. pneumoniae *(8; 14.5)	62.5		37.5	62.5		37.5	62.5	12.5	25.0	62.5		37.5	75.0	12.5	12.5	75.0		25.0	75.0		25.0
	*E. cloacae *(7; 12.7)	42.9		57.1	42.9		57.1	42.9		57.1	14.3	14.3	71.4	100			100			100		
	*P. mirabilis *(2; 3.6)	100			100			100			100			50.0	50.0		100			100		
	*M. morganii *(1; 1.8)	100			100			100			100			100			100			100		
																					
Burns (15)	*E. coli *(3; 20.0)	66.7		33.3	66.7		33.3	66.7		33.3	66.7		33.3	100			100			100		
	*K. pneumoniae *(11; 73.3)	9.1		90.9	9.1		90.9	9.1		90.9	63.6	18.2	18.2	72.7		27.3	36.4	18.2	45.5	63.6	9.1	27.3
	*P. vulgaris *(1; 6.7)	100			100			100			100			100			100			100		
																					
Pediatric (19)	*E. coli *(11; 57.9)	72.7		27.3	72.7		27.3	90.9	9.1		100			100			100			100		
	*K. pneumoniae *(3; 15.8)	66.7		33.3	100			66.7		33.3	100			100			100			100		
	*E. cloacae *(1; 5.3)	100			100			100					100	100			100			100		
	*K. oxytoca *(1; 5.3)	100			100			100			100			100			100			100		

	*S. flexneri *(1; 5.3)			100			100	100			100			100			100			100		
	*S.sonnei *(2; 10.5)			100			100		100		100			100			100			100		
																						

CTX, cefotaxime; CTR, ceftriaxone; CAZ, ceftazidime; CFO, cefoxitin; IMP, imipenem; ETP, ertapenem; MRP, meropenem; S, sensitive; R, resistant; I, intermediate

**Table 2:** Antimicrobial susceptibility patterns of β-lactamase-producing *Enterobacteriaceae*

		Resistance to antimicrobial agents (%)																							
β-lactamases	Isolates (No/total; %)	FEP			Amp			AMC			PIZ			SXT			Am			Gen			CIP		
		S	I	R	S	I	R	S	I	R	S	I	R	S	I	R	S	I	R	S	I	R	S	I	R
ESBL	*E. coli *(75/219; 34.2)	52.0	14.7	33.3			100	82.7	14.7	2.7	90.7	6.7	2.7	24.0		76.0	98.7		1.3	50.7	1.3	48.0	21.3	5.3	73.3
	*K. pneumoniae * (37/57; 64.9)	18.9	18.9	62.2			100	24.3	21.6	54.1	29.7	13.5	56.8	21.6		78.4	56.8	16.2	27.0	24.3		75.7	35.1	10.8	54.1
	*S. flexneri* (2/2; 100)	100					100	100			100					100	100			50.0		50.0	100		
	*S. sonnei *(2/2; 100)	100					100	100			100					100	100			50.0		50.0	50.0		50.0
																								
AmpC	*E. coli *(14/219; 6.4)	78.6		21.4	7.1		92.9	14.3		85.7	57.1	21.4	21.4	42.9		57.1	92.9		7.1	78.6		21.4	14.3	7.1	78.6
	*E. cloacae* (9/14; 64.3)	88.9		11.1	11.1		88.9		22.2	77.8	100			66.7		33.3	100			88.9		11.1	55.6		44.4
	*P. mirabilis *(2/5; 40.0)	100			50.0		50.0	100			100			50.0		50.0	100			50.0	50.0		100		
	*S. marcescens *(1/1; 100)	100			100			100			100			100			100			100			100		
	*M. morganii *(2/2; 100)	100			100			100			100			100			100			100			100		
																								
ESBL+ AmpC	*E. coli *(9/219; 4.1)	33.3	22.2	44.4			100		66.7	33.3	22.2	66.7	11.1	11.1		88.9	100			66.7		33.3	11.1		88.9
	*E. cloacae* (5/14; 35.7)	40.0		60.0			100	20.0		80.0	100					100	100					100	40.0	40.0	20.0
	*C. freundii *(1/1; 100)	100					100			100	100			100			100			100			100		
																								
MBL	*K. pneumoniae *(9/57; 15.8)			100			100		11.1	88.9			100			100	11.1		88.9			100	11.1		88.9
KPC	*K. pneumoniae *(4/57; 7.0)		25.0	75.0			100			100			100	50.0		50.0	75.0		25.0			100	50.0		50.0
OXA-48	*K. pneumoniae *(2/57; 3.5)			100			100			100			100			100		50.0	50.0			100	50.0		50.0
																									
ESBL+ Decreased permeability	*E. coli *(2/219; 0.9)		50.0	50.0			100	100			50.0		50.0			100	100			50.0		50.0	100		
	*K. pneumoniae * (5/57; 8.8)	20.0	40.0	40.0			100			100			100			100	60.0	40.0		40.0		60.0	60.0		40.0
																								
AmpC+ Decreased permeability	*E. coli *(4/219; 1.8)	50.0	25.0	25.0			100	25.0		75.0	25.0	25.0	50.0	25.0		75.0	100			50.0		50.0	25.0		75.0
	*E. cloacae* (1/14; 7.1)	100					100		100		100			100			100			100					100
	*P. mirabilis *(2/5; 40.0)	100			50.0		50.0	100			100			50.0		50.0	100			50.0	50.0		100		

FEP, cefepime; Amp, ampicillin; AMC, amoxicillin/clavulanate; PIZ, piperacillin/tazobactam; SXT, terimethoprim/sulfamethoxazole; Am, amikacin; Gen, gentamicin; CIP, Ciprofloxacin; S, sensitive; R, resistant; I, intermediate.

AmpCs with hydrolyzing activity against narrow, broad and extended-spectrum cephalosporins and cephamycins have been described in many Gram-negative bacilli and their plasmid-mediated types are particularly associated with multidrug resistant. Scarce information are available about the accurate prevalence of these β-lactamases due to the lack of appropriate diagnostic tests, but it appears that the incidence is less than ESBLs (2). According to the results of phenotypic confirmatory tests performed on cefoxitin non-susceptible isolates, 14% of all isolates including *E. coli* (10.5%),* and E. cloacae* (100%) had detectable AmpC activities. The overall prevalence of AmpCs reported in our study is in complete agreement with a study from India which reported the overall prevalence of 12.5% among *Enterobacteriaceae* (26). Also we found 16 cefoxitin non-susceptible strains out of 57(28%) *K. pneumoniae* isolates with negative phenotypic tests for AmpC production. This rate for *E. coli* was 29 non-susceptible strains out of 219(13.2%) from which 23(10.5%) strains were AmpC positive. Although these results may indicate the high occurrence of AmpC-producing *E. coli* in comparison to other similar studies (27-28), the actual incidence of AmpCs remains unknown due to the inability of current phenotypic tests to accurately detect the plasmid-mediated AmpCs (29). Moreover, sizable number of cefoxitin resistant isolates with negative AmpC confirmatory test or negative PCR previously reported by Manoharan *et al* (26). Cefoxitin resistant isolates with negative AmpC confirmatory test may be due to existence of other unknown mechanisms for this phenomenon.

Emergence of CPE in worldwide is nowadays a main public health concern because of limited therapeutic options and high mortality rate associated with invasive infections due to these isolates. Carbapenemases are diverse versatile β-lactamases with variable hydrolyzing activities against carbapenems and other β-lactam drugs and are often associated with extensive or pan-resistance to several classes of antimicrobials. Until recently, the members of family *Enterobactericeae* did not have any significant mechanism for carbapenem resistance but the acquisition of transmissible carbapenemase genes from more resistant organisms such as Pseudomonas and Acinetobacter resulted in that CPE have been recently drawn widespread attention (30). In our study, carbapenemases were detected only in *K. pneumoniae* isolates (26.3%). These results are in agreement with the fact that carbapenamases in *Enterobacteriacae* are primarily found in *K. pneumoniae*, and to a much lower prevalence in *E. coli* and other members of *Enterobacteriacae* family (31). This study shows that the occurrence of CPE is high in East Azerbaijan province of Iran and is comparable to other endemic parts of the world. These results are in consistent with data reported in current studies suggesting that carbapenemases mainly appeared in Asia (31). According to our results, Sina and Imam Reza hospitals of Tabriz are the endemic foci of CPEs specially KPC and MBL types similar to other part of the world such as Italy and Greece where KPC and MBL are endemic, respectively (22). Both Sina and Imam Reza hospitals are main educational medical centers of East Azerbaijan province of Iran, receiving diverse populations of complicated patients from surrounding provinces and neighboring republic of Azerbaijan. 

According to the antimicrobial susceptibility results, imipenem, meropenem and ertapenem were the most active antibiotics against all isolates particularly all ESBL/AmpC-producing strains. These results are compatible with studies in Turkey and Canada reporting 100%, 100% and 98.0% susceptibilities toward imipenem, meropenem, and ertapenem, respectively (23, 28 and 32). In this study, susceptibility to imipenem, meropenem and ertapenem among ESBL-producing *K. pneumoniae* isolates were 66.7%, 66.7% and 75.4% respectively. Comparing this values with 100% susceptibility to imipenem among inpatient isolates of *K. pneumoniae* reported from a study in Tehran and 100% susceptibility to imipenem and meropenem and also 94% susceptibility to ertapenem reported from Istanbul, explains higher occurrence of carbapenemase-producing *K. pneumoniae* strains in East Azerbaijan province compared to Tehran and Istanbul (32-33). In addition, the majority of carbapenemase-producing *K. pneumoniae* isolates (13 out of 15 strains) were resistant to all non-β-lactam antibiotics tested in this study. This is consistent with the fact that many carbapenemase producers carry resistance determinants for structurally unrelated antibacterial drugs (31).

Based on the *in-vitro* antimicrobial susceptibility patterns observed in this study, amikacin, piperacillin/tazobactam and amoxicillin/clavulanic acid were second the most potent drugs after carbapenems against ESBL/AmpC-producing *E. coli* strains with susceptibility rates of 98%, 78.8% and 64.6%, respectively. Gentamicin and cefepime showed moderate activities against these strains with 55.6% and 54.5% susceptibility rates. The highest rates of resistance were observed for ampicillin (99%), ciprofloxacin (80.8%) and co-trimoxazole (74.7%) among ESBL positive *E. coli* strains. Also, amikacin was found to be second the most active antibiotic against ESBL-producing *K. pneumoniae* with moderate rate of susceptibility reaching 53.8% on disc diffusion test. Other drugs displayed poor activities among ESBL-producing *K. pneumoniae* with susceptibility rates ranging from 0.0% for ampicillin to 33.3% for ciprofloxacin. 

In the present study, the rate of multidrug resistant β-lactamase-producing *E. coli* and *K. pneumoniae* was 93.9% and 100%, respectively (P < 0.05). These values was significantly higher than those from annual Canadian Ward surveillance study (CANWARD) reporting 83.3% and 31% MDR rates among ESBL and AmpC-producing *E. coli* isolates (28). This high rate of MDR among β-lactamase-producing isolates explains the high proportion and horizontal dissemination of ESBL, AmpC, and carbapenemase-encoding transferable genetic elements carrying diverse and large number of resistance genes among members of *Enterobaceriacae* due to the high selection pressure of resistance to non-β-lactam drugs (34, 35).

## Conclusions

In conclusion, the rates of ESBLs, AmpCs, carbapenemases, and MDR among *Enterobacteriaceae* isolates are high. Imipenem and meropenem still show potent antibacterial activities against all MDR β-lactamase-producers except for carbapenemase producers particularly KPC and MBL-producing *K. pneumoniae* isolates. Therefore, continuous surveillance programs, molecular characterization of carbapenemase producers and regular publication of antimicrobial susceptibility patterns can be effective tools to detect, monitor, and to successfully control CPE.
